# A Study of Thyroid Fine Needle Aspiration of Follicular Adenoma in the “Atypia of Undetermined Significance” Bethesda Category Using Digital Image Analysis

**DOI:** 10.1016/j.jpi.2022.100004

**Published:** 2022-01-20

**Authors:** Keluo Yao, Xin Jing, Jerome Cheng, Ulysses G. J. Balis, Liron Pantanowitz, Madelyn Lew

**Affiliations:** a City of Hope National Medical Center, Department of Pathology, Bellaire, Texas, USA; b Michigan Medicine, University of Michigan, Department of Pathology, Ann Arbor, MI, USA

**Keywords:** Thyroid, Fine needle aspiration, Digital image analysis, ThinPrep, Bethesda

## Abstract

**Background:**

Originally designed for computerized image analysis, ThinPrep is underutilized in that role outside gynecological cytology. It can be used to address the inter/intra-observer variability in the evaluation of thyroid fine needle aspiration (TFNA) biopsy and help pathologists to gain additional insight into thyroid cytomorphology.

**Methods:**

We designed and validated a feature engineering and supervised machine learning-based digital image analysis method using ImageJ and Python scikit-learn . The method was trained and validated from 400 low power (100x) and 400 high power (400x) images generated from 40 TFNA cases.

**Result:**

The area under the curve (AUC) for receiver operating characteristics (ROC) is 0.75 (0.74–0.82) for model based from low-power images and 0.74 (0.69–0.79) for the model based from high-power images. Cytomorphologic features were synthesized using feature engineering and when performed in isolation, they achieved AUC of 0.71 (0.64–0.77) for chromatin, 0.70 (0.64–0.73) for cellularity, 0.65 (0.60–0.69) for cytoarchitecture, 0.57 (0.51–0.61) for nuclear size, and 0.63 (0.57–0.68) for nuclear shape.

**Conclusion:**

Our study proves that ThinPrep is an excellent preparation method for digital image analysis of thyroid cytomorphology. It can be used to quantitatively harvest morphologic information for diagnostic purpose.

## List of Abbreviations

**Table:** 

AUC	Area under the curve
AUS	Atypia of undetermined significance
DIA	Digital image analysis
ETC	Extra tree classifier
FLUS	Follicular lesion of undetermined significance
FNA	Fine needle aspiration
GBC	Gradient boost classifier
ROC	Receiver operating characteristics
SQL	Structured query language
TFNA	Thyroid fine needle aspiration

## Introduction

As one of the more accessible organs for fine needle aspiration (FNA) biopsy, thyroid nodules are frequently evaluated for cytologic diagnosis to determine surgical versus conservative management. While a subset of thyroid FNA (T-FNA) contains clear cytomorphologic features of neoplastic lesions that can be definitively and reliably diagnosed amongst cytopathologists, up to 21% of cases within some institutions can display cellular and architectural atypia insufficient for definitive diagnosis, leaving a significant element of uncertainty of appropriate management for clinicians to pursue.^1^^,^^2^ Many indeterminate results due to architectural atypia identified within T-FNAs are reported by pathologists as “atypia of undetermined significance/follicular lesion of undetermined significance (AUS/FLUS)” (Bethesda category III) in the Bethesda System for Reporting Thyroid Cytopathology TBSRTC. While TBSRTC recommends molecular assays for both categories to guide management,^3^ many clinicians are seeking lower cost options to enhance the diagnostic accuracy of the existing cytological material, particularly in the indeterminate diagnostic categories.

 In our current study, we evaluated an alternative pathway to an objective, reproducible diagnosis by utilizing an existing cytologic preparation technique optimized for digital pathology and machine learning algorithms.^4^^,^^5^ The use of this technology can provide a substitute pathway to resolve indeterminate diagnostic categories through digital evaluation and classification of cytomorphologic features (follicular group architecture, smear cellularity, amount of colloid, and cytologic atypia) associated with follicular neoplasms.^6^ To our best knowledge to date, ThinPrep ® is underutilized in this regard but is widely used by many institutions for the evaluation of thyroid aspirate material. ThinPrep ® is conveniently primed for digital image analysis (DIA), as it is created to reduce the variability of stains and was originally developed for the ThinPrep Imaging System.^7^ In this study, we aim to evaluate the feasibility of applying DIA on T-FNA material prepared by the ThinPrep ® procedure and use it to gain more insight to improve the diagnostic accuracy of thyroid aspiration cytology.

## Method

### Case Collection and Image Capture

To reduce the complexity of the study, we decided to focus on the morphologic difference between surgically verified benign thyroid vs. thyroid with follicular adenoma, as the extent and degree of morphologic criteria are more subjective rendering less reproducible diagnoses in comparison to other thyroid lesions with cytologic (nuclear) atypia such as papillary thyroid lesions.

From our laboratory information system (LIS), we performed a structured query language (SQL) search for all surgical resection cases diagnosed as follicular adenoma or thyroid with nodular hyperplasia. Cross referencing the prior T-FNAs , we identified 20 T-FNAs diagnosed as AUS/FLUS, with subsequent diagnoses of follicular adenoma on surgical resections and 20 T-FNAs with subsequent diagnoses of benign thyroid nodules on surgical resections. Digital images of 10 mid-power (100x) and 10 high-power (400x) fields on the ThinPrep material were obtained using a DP71 camera (3500 Corporate Parkway, Center Valley, PA 18034, Olympus, USA) on an Olympus BX51 microscope with CellSens Entry v1.12 (Olympus, USA). The mid-power fields were randomly taken to evaluate overall specimen cellularity while the high-power fields captured follicular cells. All images associated with each case were grouped together and further reviewed by a board-certified cytopathologist (ML) to evaluate for adequate cellularity and to render a diagnosis within the Bethesda classification system. Unsatisfactory cases with insufficient cellularity were removed from the study. In total, we curated 800 images through the above process.

### Image Analysis

To maximize the use of the images, a custom image analysis algorithm was developed based on cytomorphology feature engineering and supervised machine learning.

### Cytomorphology Feature Engineering

**Figure 1:**
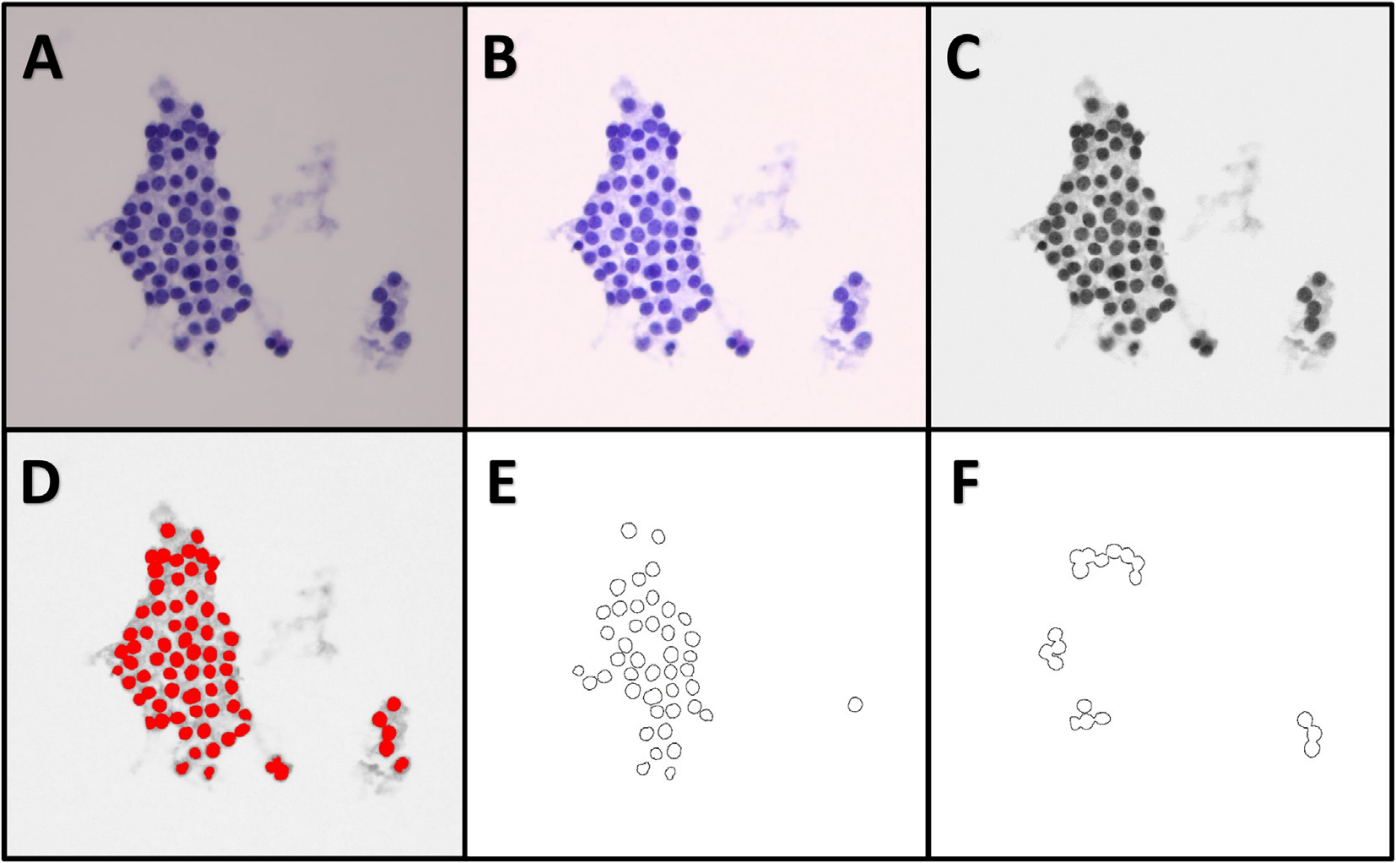
The segmentation and feature extraction of each image (A) starts with background subtraction (B), followed by conversion to 8-bit grayscale image (green channel only) through color deconvolution (C), automatic threshold segmentation, and finally a mask (red) for the nuclear features (D). The extracted nuclei features are further “gated” (high-power only) using size and circularity to separate out individual nuclei from closely grouped clusters.

We used ImageJ v1.51p (NIH, USA) to develop cytomorphology feature engineering. The process consists of image segmentation followed by feature extraction (Fig. 1). For image segmentation, we started with preprocessing of the images by substracting the background, followed by red‑green‑blue color channel separation. We only extracted the green channels and created masks for all nuclei using an automatic threshold method. The feature extraction processed focused on the nuclei which were treated as individual “particles” with low-level features. The low-level features are selectively grouped together based on the authors’ cytomorphology knowledge to form medium and high level features (Table 1). For example, a medium level feature, nuclear size, or simply size, is composed of mean and standard deviation of nuclear area, which are low level features. Cytology, a high-level feature, is composed of three medium level features, chromatin, shape, and size. For the high-power images, the “particles” were filtered by some low level features such as size and circularity to remove background noise. These low level features were also used to distinguish or “gate” individual nuclei from closely grouped clusters to detect crowding of follicular cells. The “cellularity” high level features were extracted only from the mid-power images. Altogether, we have a total of 86 low level nuclear features used to construct three medium level feature models (chromatin, shape, and size), three high level feature models (cellularity, architecture, and cytology), and two models based on magnification (low and high power).

**Table 1:** Details of the predictive models and features performance.

Magnification^1^	High^2^	Medium^3^	Low^4^	Gate^5^	Mean/StdDv^6^	Feature importance^7^	FA value^8^	B9 value^9^	T-test score^10^
Mid-power	Cellularity	N/A	Count^I^	None	Mean	0.143	268.57	164.31	0.000
Mid-power	Cellularity	N/A	Total Area^II^	None	Mean	0.104	41191.52	24885.81	0.001
Mid-power	N/A	N/A	Area^III^	None	Mean	0.060	153.44	129.37	0.122
Mid-power	N/A	N/A	Circ^IV^	None	Mean	0.083	0.90	0.90	0.832
Mid-power	N/A	N/A	MaxFeret^V^	None	Mean	0.067	12.84	12.27	0.248
Mid-power	N/A	N/A	IntDen^VI^	None	Mean	0.056	25581.13	21781.70	0.175
Mid-power	N/A	N/A	Kurt^VII^	None	Mean	0.066	-1.03	-1.02	0.586
Mid-power	N/A	N/A	Mean^VIII^	None	Mean	0.061	174.81	177.03	0.263
Mid-power	N/A	N/A	Median^IX^	None	Mean	0.057	176.21	178.17	0.329
Mid-power	N/A	N/A	MinFeret^X^	None	Mean	0.067	8.59	8.25	0.258
Mid-power	N/A	N/A	Mode^XI^	None	Mean	0.061	177.56	179.19	0.465
Mid-power	N/A	N/A	Perim^XII^	None	Mean	0.062	35.12	33.14	0.180
Mid-power	N/A	N/A	Skew^XIII^	None	Mean	0.055	-0.28	-0.26	0.050
Mid-power	N/A	N/A	Solidity^XIV^	None	Mean	0.058	0.89	0.89	0.519
High-power	Architecture	N/A	AR^XV^	Cluster	StdDv	0.010	0.38	0.30	0.017
High-power	Architecture	N/A	AR	Cluster	Mean	0.009	1.74	1.46	0.001
High-power	Architecture	N/A	Area	Cluster	Mean	0.014	5711.59	2252.09	0.000
High-power	Architecture	N/A	Area	Cluster	StdDv	0.009	4731.23	1443.71	0.000
High-power	Architecture	N/A	Circ	Cluster	StdDv	0.014	0.11	0.07	0.000
High-power	Architecture	N/A	Circ	Cluster	Mean	0.009	0.32	0.27	0.003
High-power	Architecture	N/A	MaxFeret	Cluster	Mean	0.016	98.85	67.44	0.000
High-power	Architecture	N/A	MaxFeret	Cluster	StdDv	0.009	40.91	18.64	0.000
High-power	Architecture	N/A	MinFeret	Cluster	Mean	0.011	60.02	40.96	0.000
High-power	Architecture	N/A	MinFeret	Cluster	StdDv	0.009	25.45	11.62	0.000
High-power	Architecture	N/A	Perim	Cluster	Mean	0.015	432.76	264.52	0.000
High-power	Architecture	N/A	Perim	Cluster	StdDv	0.010	274.11	108.59	0.000
High-power	Architecture	N/A	Round	Cluster	Mean	0.012	0.52	0.44	0.001
High-power	Architecture	N/A	Round	Cluster	StdDv	0.008	0.11	0.09	0.004
High-power	Architecture	N/A	Solidity	Cluster	Mean	0.015	0.69	0.57	0.000
High-power	Architecture	N/A	Solidity	Cluster	StdDv	0.012	0.06	0.04	0.000
High-power	Cytology	Chromatin	IntDen	Single	StdDv	0.020	17007.18	12785.69	0.000
High-power	Cytology	Chromatin	IntDen	Cluster	Mean	0.016	478875.76	169419.42	0.000
High-power	Cytology	Chromatin	IntDen	Single	Mean	0.016	40865.41	34437.49	0.000
High-power	Cytology	Chromatin	IntDen	Cluster	StdDv	0.010	395319.99	139400.12	0.004
High-power	Cytology	Chromatin	Kurt	Single	StdDv	0.013	0.47	0.43	0.121
High-power	Cytology	Chromatin	Kurt	Cluster	Mean	0.013	-0.48	-0.38	0.046
High-power	Cytology	Chromatin	Kurt	Single	Mean	0.012	-0.51	-0.46	0.079
High-power	Cytology	Chromatin	Kurt	Cluster	StdDv	0.008	0.38	0.30	0.126
High-power	Cytology	Chromatin	Max^XVI^	Single	StdDv	0.028	4.55	2.96	0.000
High-power	Cytology	Chromatin	Max	Single	Mean	0.020	127.32	115.62	0.001
High-power	Cytology	Chromatin	Max	Cluster	Mean	0.015	132.70	99.76	0.000
High-power	Cytology	Chromatin	Max	Cluster	StdDv	0.008	11.49	7.99	0.010
High-power	Cytology	Chromatin	Mean	Cluster	Mean	0.018	76.34	56.04	0.000
High-power	Cytology	Chromatin	Mean	Cluster	StdDv	0.016	6.67	3.76	0.000
High-power	Cytology	Chromatin	Mean	Single	StdDv	0.014	11.75	9.78	0.000
High-power	Cytology	Chromatin	Mean	Single	Mean	0.012	82.00	73.23	0.001
High-power	Cytology	Chromatin	Median^XVII^	Cluster	StdDv	0.019	8.29	4.69	0.000
High-power	Cytology	Chromatin	Median	Cluster	Mean	0.015	75.08	54.08	0.000
High-power	Cytology	Chromatin	Median	Single	StdDv	0.015	14.33	11.99	0.000
High-power	Cytology	Chromatin	Median	Single	Mean	0.013	78.86	69.88	0.001
High-power	Cytology	Chromatin	Min^XVIII^	Single	StdDv	0.015	14.14	11.14	0.001
High-power	Cytology	Chromatin	Min	Single	Mean	0.014	43.59	40.29	0.108
High-power	Cytology	Chromatin	Min	Cluster	Mean	0.012	28.39	22.31	0.001
High-power	Cytology	Chromatin	Min	Cluster	StdDv	0.009	7.88	4.86	0.000
High-power	Cytology	Chromatin	Mode^XIX^	Cluster	Mean	0.019	70.14	47.87	0.000
High-power	Cytology	Chromatin	Mode	Cluster	StdDv	0.018	14.81	9.01	0.000
High-power	Cytology	Chromatin	Mode	Single	StdDv	0.014	20.49	16.81	0.000
High-power	Cytology	Chromatin	Mode	Single	Mean	0.012	71.10	62.52	0.001
High-power	Cytology	Chromatin	RawIntDen	Single	Mean	0.018	40865.41	34437.49	0.000
High-power	Cytology	Chromatin	RawIntDen	Single	StdDv	0.016	17007.18	12785.69	0.000
High-power	Cytology	Chromatin	RawIntDen	Cluster	Mean	0.015	478875.76	169419.42	0.000
High-power	Cytology	Chromatin	RawIntDen	Cluster	StdDv	0.009	395319.99	139400.12	0.004
High-power	Cytology	Chromatin	Skew	Single	Mean	0.021	0.34	0.47	0.000
High-power	Cytology	Chromatin	Skew	Cluster	Mean	0.015	0.14	0.25	0.006
High-power	Cytology	Chromatin	Skew	Single	StdDv	0.010	0.39	0.37	0.089
High-power	Cytology	Chromatin	Skew	Cluster	StdDv	0.010	0.26	0.17	0.000
High-power	Cytology	Chromatin	StdDev	Single	Mean	0.023	20.07	18.20	0.002
High-power	Cytology	Chromatin	StdDev	Single	StdDv	0.012	4.58	3.49	0.000
High-power	Cytology	Chromatin	StdDev	Cluster	Mean	0.011	21.90	16.84	0.000
High-power	Cytology	Chromatin	StdDev	Cluster	StdDv	0.011	2.88	1.91	0.000
High-power	Cytology	Shape	AR	Single	Mean	0.017	1.31	1.34	0.038
High-power	Cytology	Shape	AR	Single	StdDv	0.012	0.27	0.28	0.163
High-power	Cytology	Shape	Circ	Single	Mean	0.022	0.73	0.76	0.004
High-power	Cytology	Shape	Circ	Single	StdDv	0.012	0.10	0.09	0.405
High-power	Cytology	Shape	MaxFeret	Single	Mean	0.016	29.13	29.01	0.804
High-power	Cytology	Shape	MaxFeret	Single	StdDv	0.012	6.20	5.77	0.064
High-power	Cytology	Shape	MinFeret	Single	Mean	0.016	22.33	21.77	0.127
High-power	Cytology	Shape	MinFeret	Single	StdDv	0.013	4.28	3.76	0.001
High-power	Cytology	Shape	Perim	Single	Mean	0.018	87.90	85.51	0.108
High-power	Cytology	Shape	Perim	Single	StdDv	0.011	19.23	17.45	0.010
High-power	Cytology	Shape	Round	Single	Mean	0.019	0.77	0.76	0.230
High-power	Cytology	Shape	Round	Single	StdDv	0.009	0.13	0.13	0.995
High-power	Cytology	Shape	Solidity	Single	Mean	0.019	0.89	0.90	0.265
High-power	Cytology	Shape	Solidity	Single	StdDv	0.011	0.03	0.03	0.371
High-power	Cytology	Size	Area	Single	Mean	0.016	482.09	461.70	0.100
High-power	Cytology	Size	Area	Single	StdDv	0.011	183.26	157.58	0.000

**1**. The **magnification** models are consisted of mid-power (100x) and high-power (400x) models. **2**. **High** = high level features; **3**. **Medium** = medium level features; **4**. **Low** = low level features. **5**. The “**gate**” filters follicular cell nuclei into single vs overlapping clusters based on: single nuclei have areas (III) between 100 and 1200 pixels and circ (IV) between 0.5 and 1.0; overlapping clusters have areas (III) between 1200 to infinite pixels and circularity between 0.0 and 1.0. **6**. Values collected as **mean vs standard deviation**. **7**. **Feature importance** dictates contribution (in percentage) of each feature to the predictive accuracy of the model. **8**. Average **value** collected from **follicular adenoma** images. **9**. Average **value** collected from **benign (B9) thyroid** images. **10**. **Student’s *T-*test***P* values of each feature based on comparing values from follicular adenoma vs benign thyroid.

**Count (I)** - Number of separated nuclei, including both single and clustered nuclei; **Total area (II)** - Total area of the image occupied by nuclei in pixels; each pixel corresponds to an area of 0.064 μM^2^ for the high-power model and 0.016 μM^2^ for the mid-power model; **Area (III)** - Area of region of individual nuclei in square pixels; **Circ (IV)** - 4 π (Area/Perimeter^2^); 1.0 is a perfect circle; the value approaches 0 as the shape elongates; **MaxFeret (V)** - Feret's diameter: Maximum caliper; conversion factor 0.08 μM for high-power model and 0.04 μM for mid-power model; **IntDen (VI)** - Integrated density: area times mean gray value; **Kurt (VII)** - Kurtosis: The fourth-order moment about the mean; **Mean (VIII)** - Average gray value of the pixels in each nucleus/cluster of nuclei; The values range from 0 to 255; **Medium (IX)** - The median gray value of the pixels in the entire image; **MinFeret (X)** - Minmum Feret's diameter: minimum caliper; conversion factor 0.08 μM for high-power model and 0.04 μM for mid-power model; **Mode (XI)** - Most frequently occurring gray value of the pixels in each nucleus/cluster of nuclei; **Perim (XII)** - The length of the outside boundary of each nucleus/cluster of nuclei; multiple the value by 0.08 to get a measurement in μM for the high-power model and 0.04 for the mid-power model; **Skew (XIII)** - The third-order moment about the mean; **Solidity (XIV)** - Area/Convex Area; **AR (XV)** - Aspect ratio: Major axis/Minor axis; **Max (XVI)** - Maximum gray values of the pixels in each nucleus/cluster of nuclei; value range from 0 to 255; **Median (XVII)** - The median value of the pixels in the entire image; values range from 0 to 255; **Min (XVIII)** - Minimum gray values of the pixels in each nucleus/cluster of nuclei; values range from 0 to 255; **Mode (XIX)** - Most frequently occurring gray value of the pixels in each nucleus/cluster of nuclei; values range from 0 to 255.

### Supervised Machine Learning

Supervised machine learning methods aim to automatically create algorithms based on known paired input (features) and expected output (e.g., ground truth) data. Training data are used to optimize the weights and parameters of the algorithms while the validation data were used to validate the generalizability and performance of the trained algorithm. Utilizing the above rules, the various combination of features based on the models were utilized as the input and surgical report (Follicular adenoma vs benign thyroid) of the T-FNA were the expected output. Follicular adenoma was considered as a positive result. Using Python sklearn library, we used gradient boost classifier (GBC) and extra tree classifier (ETC) as our supervised machine learning methods. The training and validation data were randomly split 1:1 from the collected data using a data splitting algorithm. The process was also repeated three times to further ensure generalization and to prevent overfitting. We also used extra tree classifier to evaluate the importance of low level features using all available data.

## Result

The measure of a predictive test performance calls for measurement in accuracy, the closeness of the measurements to a specific value; precision, also known as positive-predictive value; recall, sensitivity. Since all features were used between the high and low power models, their performances are the direct measurement of the DIA algorithm design. Using validation data only, the mid-power model achieved an average accuracy of 0.71 (0.70–0.74), precision 0.72 (0.69–0.74), and recall 0.71 (0.64–0.75); the high-power model achieved an average accuracy of 0.67 (0.63–0.72), precision of 0.67 (0.62–0.74), and recall 0.69 (0.60–0.74). By direct comparison, the cytopathologist who reviewed all the images achieved an accuracy of 0.625, precision 0.57, and recall 0.95.

**Figure 2:**
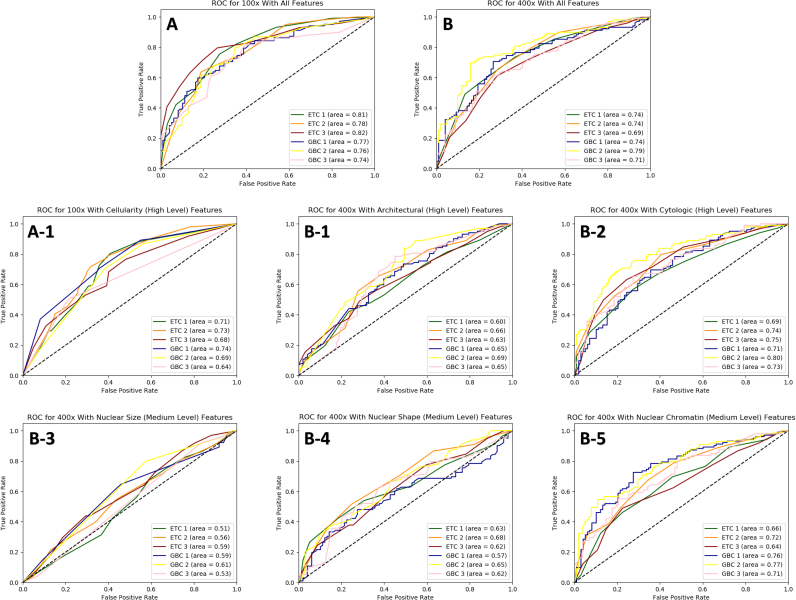
The predictive performance evaluation of mid-power (A) and high-power (B) models, high level features (A-1, B-1, B-2), and medium level features (B-3, B-4, B-5) using receiver operating characteristics (ROC) and quantified by area under the curve (AUC); ETC = extra tree classifier; GBC = gradient boost classifier.

Receiver operating characteristics (ROC) is also used to evaluate the diagnostic ability of a test as its discrimination threshold is changed. Using validation data only, the AUCs are 0.75 (0.74–0.82) for mid-power magnification model and 0.74 (0.69–0.79) for high-power magnification model. For high level features models, AUCs are 0.70 (0.64–0.73) for cellularity, 0.65 (0.60–0.69) for architecture, and 0.74 (0.69–0.80) for cytology. The AUC for ROC for medium level features are 0.57 (0.51–0.61) for nuclear size, 0.63 (0.57–0.68) for nuclear shape, and 0.71 (0.64–0.77) for nuclear chromatin (Fig. 2). Table 1 gives additional details on the breakdown of prediction accuracy contribution and statistical analyses of all features.

Since the high- and medium-power magnification models have reasonable performance base on the validation results, the high and medium level features models can be considered as statistical hypothesis tests to evaluate the importance of each group of features and their contribution to the accuracy of the models. Based on this method, all three high level features, cellularity, architecture, and cytology appear to contribute significantly. For the medium level features, nuclear chromatin appears to be the strongest contributor while nuclear shape is a distant second. The nuclei size, on the other hand, appears to be non-contributory , a finding collaborated by statistical analysis (*P*=0.10) of the size variation between T-FNA from follicular adenoma and benign thyroid(Table 1).

The minimal presence of colloid material in ThinPrep combined with technical limitations prevented the incorporation of these morphologic features into our models.

## Discussion

The current evaluation of T-FNA relies on manual visual evaluation by cytopathologists. It is known that while the human visual system is excellent at recognizing patterns, it performs poorly on quantitative tasks and is susceptible to optical illusions.^8^ Most suspicious or malignant (Bethesda category IV to VI) T-FNA cases show higher rates of diagnostic reproducibility among cytopathologists as they present with more pronounced architectural and cytologic features. In these cases, there is little need for repeated T-FNA or ancillary molecular tests for further characterization as the evidence for surgical management is well established. However, for Bethesda category III, the degree of cytologic and architectural atypia may be subtle and variable, which explains the high degree of inter-observer variability.^9^ Furthermore, the Bethesda criteria for this diagnostic category, whether architectural or cytologic atypia, are not defined in quantifiable methods and therefore are fundamentally subjective.

Our study shows that while looking at the exact same set of images, board-certified cytopathologists may err on the side of caution and sacrifice overall accuracy. While molecular testing provides an alternative to repeat T-FNA , it comes at a cost of additional needle passes and the assay itself.^10^ Additionally, their exact predictive performance for entities like follicular adenoma remains controversial based on existing published data.^11^ Our results show that routine T-FNA augmented by using ThinPrep material can produce predictions with the pre-existing diagnostic material with increased overall accuracy by quantitatively evaluating morphologic features. Therefore, concurrent evaluation of preliminarily indeterminate T-FNA with DIA may present as a more cost-effective method for evaluating thyroid nodules without additional biopies or molecular studies. Additionally, as a liquid-based cytology preparation that uses standardized instruments to produce monolayers of well-stained and well-preserved cells, ThinPrep may be further explored for further non-gynecologic image analysis applications.

Our DIA design also examines the morphologic difference between T-FNA from follicular adenoma and benign thyroid. The performances of the high level feature models show cellularity, architecture, and cytology appear to contribute to the accuracy of the models (Table 1 and Fig. 2). ThinPrep material from follicular adenoma has a higher degree of cellularity, greater follicular cell crowding, and quantifiable nuclear difference than benign thyroid (Table 1 and Fig. 2). Further characterization of the nuclear morphology profiles using the medium level features shows nuclear chromatin appears to be the strongest contributor to accuracy while the nuclear shape is a distant second. The nuclear size was not a discriminating feature (with AUC close to 0.5) and this finding is further supported by the student *T*-test (*P* > 0.05) for nuclear size (Table 1). Characterization of the nuclear chromatin profile and shape difference beyond the listed performance and statistical metrics is suboptimal due to limitations of sample size and technical limitations (Table 1). However, the above findings reaffirm that cellularity, chromatin texture, and architectural features are diagnostically important in ThinPrep-based T-FNA for follicular adenoma.

To the best of our knowledge, this is the first attempt to apply DIA to simultaneously build predictive models to better separate indeterminate thyroid diagnostic categories (Bethesda III) and to investigate T-FNA cytomorphology in ThinPrep material. While T-FNA cytomorphology is well studied on manually made smears, the decades of utilization in computer image analysis assisted diagnosis for gynecologic cytology (e. g., ThinPrep Imaging System) and the recent advances in digital image analysis merit a second look for expanded applications for liquid-based preparation such as ThinPrep.^12^

Limitations of our current study include the low number of cases in the dataset and comparing DIA against a single cytopathologist. The scope of the DIA algorithm is currently limited to T-FNA of follicular adenoma or benign thyroid nodule with ThinPrep material. A whole slide imaging method was not used due to limited development time. We do believe that mid- and high-power models can sufficiently capture the vast majority of the morphologic features and thus this study can serve as proof-of-concept and pave ways for more advanced future studies to build DIA-based decision-support tools for T-FNA .
