# Erratum to “A study of thyroid fine needle aspiration of follicular adenoma in the ‘atypia of undetermined significance’ Bethesda category” [Journal of Pathology Informatics Volume 13, 2022, 100004]

**DOI:** 10.1016/j.jpi.2025.100429

**Published:** 2025-04-29

**Authors:** Keluo Yao, Xin Jing, Jerome Cheng, Ulysses G.J. Balis, Liron Pantanowitz, Madelyn Lew

**Affiliations:** a City of Hope National Medical Center, Department of Pathology, Bellaire, TX, USA; b Michigan Medicine, University of Michigan, Department of Pathology, Ann Arbor, MI, USA

The publisher regrets that the Declaration of Competing Interests was missing in the published article. The statement should read as follows:

## Declaration of competing interest

The authors declare the following financial interests/personal relationships which may be considered as potential competing interests:

Keluo Yao reports was provided by 10.13039/100013015Cedars-Sinai Medical Center. Keluo Yao reports a relationship with CEDARS SINAI that includes: employment. If there are other authors, they declare that they have no known competing financial interests or personal relationships that could have appeared to influence the work reported in this paper.

The publisher would like to apologise for any inconvenience caused.

